# Day One Cell-Free DNA Levels as an Objective Prognostic Marker of Mortality in Major Burns Patients

**DOI:** 10.3390/cells14110821

**Published:** 2025-06-01

**Authors:** Sebastian Tullie, Ali Asiri, Animesh Acharjee, Naiem S. Moiemen, Janet M. Lord, Paul Harrison, Jon Hazeldine

**Affiliations:** 1 Department of Inflammation and Ageing, School of Infection, Inflammation and Immunology, College of Medicine and Health, University of Birmingham, Birmingham B15 2TT, UKnaiem.moiemen2@nhs.net (N.S.M.);; 2 University Hospitals Birmingham National Health Service (NHS) Foundation Trust, Queen Elizabeth Hospital Birmingham, Birmingham B15 2GW, UK; 3 Birmingham Women’s and Children’s NHS Foundation Trust, Birmingham Children’s Hospital, Birmingham B4 6NH, UK; 4 Institute of Cancer and Genomic Sciences, University of Birmingham, Birmingham B15 2TT, UK; a.acharjee@bham.ac.uk; 5 The Scar Free Foundation Centre for Conflict Wound Research, Queen Elizabeth Hospital Birmingham, Birmingham B15 2GW, UK; 6 National Institute for Health Research Surgical Reconstruction and Microbiology Research Centre, Queen Elizabeth Hospital Birmingham, Birmingham B15 2TT, UK; 7 Medical Research Council (MRC) Versus Arthritis, Centre for Musculoskeletal Ageing Research, University of Birmingham, Birmingham B15 2TH, UK

**Keywords:** burns, cell-free DNA, inflammation, mortality, thermal injury

## Abstract

Background: Cell-free DNA (cfDNA) released during tissue damage has attracted interest as a marker of patient outcomes. However, limited research has examined its predictive utility in thermally injured patients. Methods: This study measured cfDNA concentrations across days 1, 3, 7, 14, and 28 post-burn in a total cohort of 98 adult patients with total body surface area (TBSA) burns ≥ 15% and healthy controls (HC). CfDNA concentrations in survivors (n = 79) versus non-survivors (n = 16) were compared and area under the receiver operating curve (AUROC) models generated to evaluate cfDNA as a predictor of mortality. Results: Patient cfDNA levels were significantly elevated at all time points compared to HC. Positive correlations were identified between day 1 cfDNA concentrations (n = 95) and %TBSA (r = 0.413, *p* < 0.0005), rBAUX (r = 0.365, *p* = 0.0005) and SOFA (r = 0.391, *p* = 0.0002). On day one, cfDNA levels showed good discriminatory ability for distinguishing between survivors and non-survivors (AUROC 0.778), with an optimal cut-off value of 446.37 pg/mL exhibiting a sensitivity of 0.80 and specificity of 0.70. Predictive models built on rBAUX, SOFA, interleukin(IL)-6 and IL-10 generated AUROC values of 0.733, 0.743, 0.472, and 0.688 respectively. Conclusions: Major burns result in immediate and persistent cfDNA elevation, with concentrations on day one higher in non-survivors. Plasma cfDNA concentrations on day one post-burn showed good performance as a prognostic marker for mortality. CfDNA therefore represents a rapid objective measure that may be useful during acute burn assessments to aid mortality predictions.

## 1. Introduction

Traumatic injuries are responsible for approximately 8% of deaths globally [1], with an estimated 180,000 deaths resulting from burns annually [2]. Though this latter mortality figure has decreased from previous reported estimates [3], major burns remain the fourth most common type of trauma [2] and represent one of the most challenging physiological insults [4]. Thermal injury triggers a systemic stress response driven by inflammation and immune dysfunction that results in the development of sepsis and multiple organ failure [5], two secondary complications that represent major causes of post-burn mortality [6,7]. Thus, the early identification of those patients at risk of poor outcomes post-burn is of the utmost clinical importance to help target therapies that will improve survival post-injury.

One endogenous factor that is elevated in the circulation of burns and trauma patients post-injury is cell-free DNA (cfDNA) [8,9]. CfDNA is defined as the circulating extracellular DNA released passively from necrotic or apoptotic cells [10], or derived from the active generation of neutrophil or monocyte extracellular traps [11,12]. In trauma patients, cfDNA concentrations have been found to be significantly elevated above the levels detected in healthy controls (HC) within minutes of injury [9,13].

CfDNA has attracted significant interest as a biomarker of post-injury effects such as systemic inflammatory response syndrome [11], sepsis [14], multiple organ failure [15], and mortality [10,16]. In trauma, the short half-life of cfDNA is thought to make it a suitable marker of patient condition in the hyper-acute phase, with early increases linked to developing coagulopathy [17] and endotheliopathy [18]. In thermal injury, cfDNA has been found to be elevated in the first 48 hours post-burn, with levels correlating positively with total body surface area (TBSA) burns [8]. Other studies in the setting of thermal injuries have quantified admission cfDNA levels and found them to be positively associated with burn depth and length of hospital stay [19,20], as well as being significantly higher in those with inhalation injuries [21] and in non-survivors [22].

CfDNA has been proposed as having value as a prognostic indicator, with elevated concentrations specifically linked to the development of sepsis and mortality amongst critically unwell ITU patients [23,24,25], as well as predicting acute lung injury and mortality post-trauma [16,26]. Alongside cfDNA, other circulating factors that have shown potential as prognostic indicators of mortality post-burn are the inflammatory cytokines interleukin (IL)-6 and IL-10 [27,28]. However, to our knowledge, no study to date has compared the utility of cfDNA and cytokine concentrations as discriminators of clinical outcomes in the same patient cohort.

To date, studies that have measured cfDNA levels in burns patients have enrolled relatively small numbers of patients, with one study explicitly detailing the need to further elucidate the role of cfDNA as a predictor of mortality in larger cohorts of burn patients [22]. Furthermore, there is a paucity of studies exploring longitudinal cfDNA trends post-burn, with only two studies measuring cfDNA concentrations beyond the first week post-injury [8,14].

Here, we measured cfDNA levels longitudinally in burns patients, across days 1 to 28 post-injury, in what is the largest reported burn patient cohort in cfDNA studies. Using these data, we set out to examine the relationship between cfDNA and clinical outcomes: specifically, mortality, length of hospital stay, and length of ITU stay. We also explored the discriminatory ability of cfDNA relative to established clinical indices (e.g., TBSA) and other inflammatory markers (IL-6 and IL-10), in order to establish its potential value as a prognostic indicator.

## 2. Materials and Methods

### 2.1. Patient Cohorts

#### 2.1.1. Burns

Data were collected from adult patients (≥16 years old) that had sustained a total body surface area (TBSA) burn ≥15%. Enrolled within 24 hours of injury, patients were recruited into the Scientific Investigation of the Biological Pathways Following Thermal Injury-2 (SIFTI-2) study; this is an ongoing prospective observational cohort study of children and adult patients with moderate and severe burn injuries. SIFTI-2 (trial registration number:NCT04693442) received ethical approval from the West Midlands, Coventry and Warwickshire Research Ethics Committee (REC reference:16/WM/0217), with details pertaining to the study design and inclusion and exclusion criteria, as well as patient consent outlined in the study protocol [29].

#### 2.1.2. Healthy Controls

A total of 25 adult volunteers (17 male, 8 female; mean age 38 years, range 22–78 years) were recruited in accordance with the ethical approval granted by the University of Birmingham Research Ethics Committee (Ref: ERN_12-1184) to serve as HCs. They were defined as individuals who were not prescribed regular medications for a diagnosed illness and who had not had an acute infection prior to enrolment.

### 2.2. Clinical Data Collection

Patient demographic and clinical data were obtained from electronic and physical medical records. The data collated included patient age, gender, time of injury and injury mechanism. Burn injury severity data included TBSA, the presence of inhalation injury, the abbreviated burn severity index (ABSI), the revised Baux score (rBAUX), the sequential organ failure assessment (SOFA) score and the Denver score. Further outcome data collected included mortality (patients who died during hospital admission), hospital-free days (30 minus days spent in hospital) [30], and ITU-free days (30 minus days spent in ITU). Patients who died in the hospital or the ITU within 30 days of admission were assigned a score of 0. Patients were also dichotomised using length of stay (LOS) data into the expected-hospital-LOS (<2 days LOS/%TBSA) group and the longer-than-expected-hospital-LOS (≥2 days LOS/%TBSA) group, as well as into extended-ITU-LOS (≥10 days) and non-extended-ITU-LOS (<10 days) groups, as per previous research [31,32].

### 2.3. Blood Sampling

Blood samples were collected into BD Vacutainer^®^ tubes (BD Biosciences, Oxford, UK) containing z-serum clotting activator or a 1/10 volume of 3.2% trisodium citrate. The data presented here from burn patients enrolled into the SIFTI-2 study were derived from the analysis of blood samples collected at five post-injury time points: days 1, 3, 7, 14, and 28. These timepoints were chosen as they cover both the acute and subacute post-burn inflammatory response, thereby providing an insight into the kinetic profile of plasma cfDNA levels over time post-burn.

### 2.4. Preparation of Platelet-Free Plasma (PFP) and Serum

Citrate anti-coagulated blood was centrifuged at 2000× *g* for 20  min at 4 °C, after which the PFP was collected and centrifuged at 13, 000× *g* for 2  min. For serum, blood samples were collected into BD Vacutainer^®^ tubes containing z-serum clotting activator and allowed to clot for 30  min at room temperature prior to centrifugation at 1620×  *g* for 10  min. Serum was collected and stored at −80 °C prior to analysis.

### 2.5. Fluorometric Analysis of Plasma Cell-Free DNA Levels

Concentrations of cfDNA were measured in duplicate via a fluorometric assay using a SYTOX^®^ Green dye (Life Technologies, Warrington, UK). Briefly, 10 μL of patient serum or PFP samples were incubated with 5  μM SYTOX^®^ Green dye for 10 min at room temperature before fluorescence was measured using a BioTek^®^ Synergy 2 fluorometric plate reader (NorthStar Scientific, Potton, UK) at 485 nm and 528 nm excitation and emission, respectively. A λ-DNA (Fisher Scientific, Loughborough, UK) standard curve was used for sample calibration.

### 2.6. Quantification of IL-6 and IL-10 Concentrations

Following the manufacturer’s guidelines, concentrations of IL-6 and IL-10 were measured in serum samples using a commercially available magnetic bead multiplex immunoassay (BioRad, Hertfordshire, UK).

### 2.7. Statistical Analyses

Analysis was performed using GraphPad PRISM software (Version 10, GraphPad Software Ltd., Boston, MA, USA) and SPSS Statistics (Version 29.0.1.1, IBM). Data distribution was assessed for normality using Kolmogorov–Smirnov or Shapiro–Wilk tests. Normally distributed data were analysed using a one-way ANOVA with a Dunnett’s or Bonferroni’s multiple comparison post hoc test, an unpaired student t-test or a paired student t-test. Non-normally distributed data were analysed using a Kruskal–Wallis test with Dunn’s multiple comparison post hoc test, Mann–Whitney U tests, Friedman one-way ANOVA, or Wilcoxon matched-pairs signed rank tests. Relationships between continuous variables were assessed using Spearman’s correlation coefficient. *p* values generated from the correlative analysis were adjusted for multiple testing using the false discovery rate correction according to the Benjamini–Hochberg method. Area under the receiver operating curve (AUROC) analyses were performed to assess the discriminatory ability of cfDNA in recorded outcomes. Statistical significance was set at a threshold of *p* ≤ 0.05. Data presented in histograms represent the mean ± standard error of mean values, with raw data points from each study participant overlaid.

## 3. Results

### 3.1. Patient Demographics

A total of 98 burns patients (77 males, 21 females) were enrolled in this study (Table 1). The patients had a mean age of 47 years (range 16–84 years) and presented with a mean TBSA burn of 35% (range 15–85%), and flame burn was the principal mechanism of injury. In total, 44.9% of patients sustained a concomitant inhalational injury, with mean hospital and ITU-free days calculated at 4 and 15, respectively. The mortality rate in this cohort was 17.3%.

Of the 98 burns patients enrolled into this study, not all had measurements of circulating cfDNA levels performed at days 1, 3, 7, 14 and 28 post-injury for several reasons, which included mortality, refusal of blood sampling, insufficient blood volumes acquired for plasma extraction, or study withdrawal (Supplementary Figure S1).

### 3.2. Thermal Injury Results in Elevated Concentrations of Circulating cfDNA

**Figure 1:**
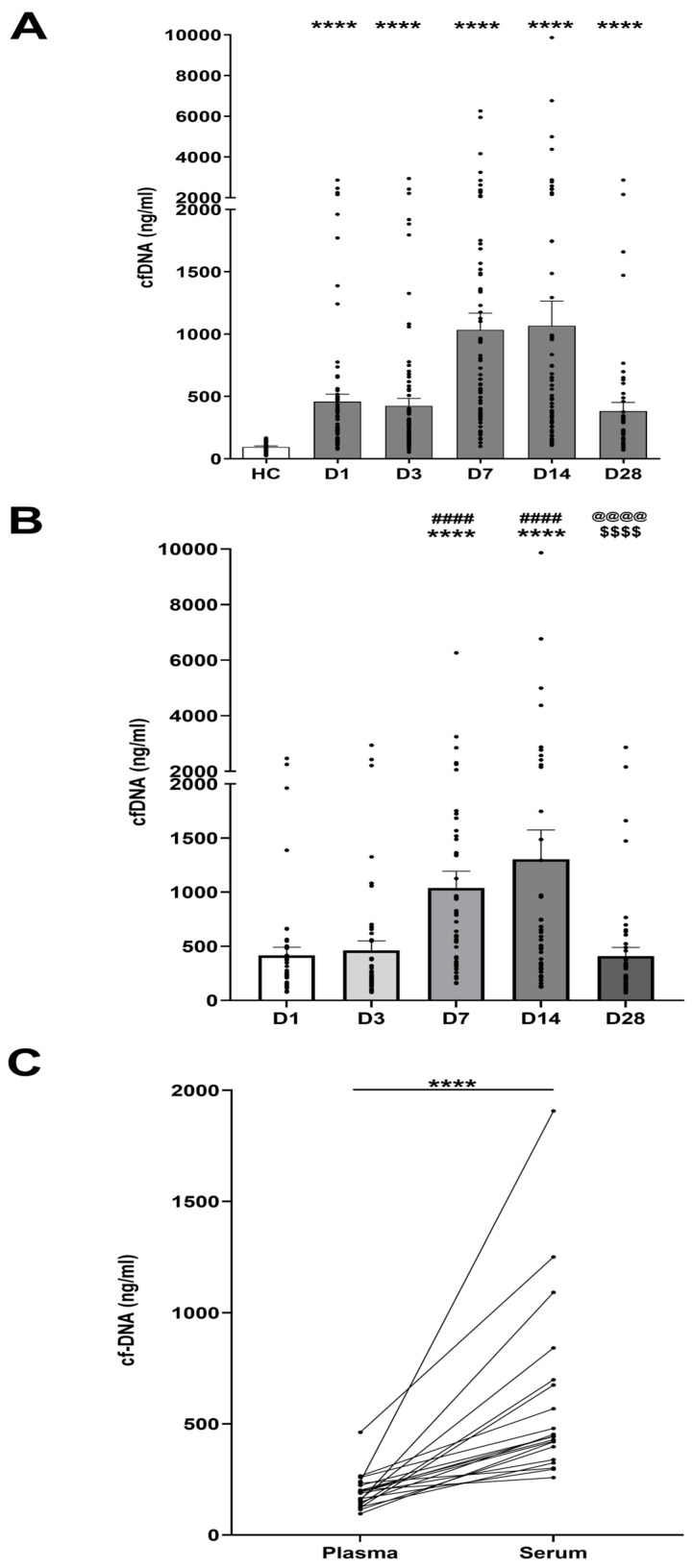
Thermal injury results in elevated circulating concentrations of cell-free DNA (cfDNA). (**A**) CfDNA concentrations measured in healthy controls (HC, n = 25) and thermally-injured patients on days 1 (D1, n = 95), 3 (D3, n = 87), 7 (D7, n = 76), 14 (D14, n = 69) and 28 (D28, n = 55) post-burn. ****p < 0.0001 vs. HC. (**B**) Repeated measures analysis of cfDNA concentrations in thermally injured patients (n = 48). **** *p* < 0.0001, D1 Vs. D7 & D14. #### *p* < 0.0001, D3 Vs. D7 & D14. $$$$ *p* < 0.0001, D7 Vs. D28. @@@@ *p* < 0.0001, D14 Vs. D28. (**C**) CfDNA concentrations measured in paired plasma and serum samples of burns patients (n = 20). **** *p* < 0.0001.

Across all sampling time-points, burns patients presented with significantly elevated cfDNA concentrations in PFP samples when compared to HC (Figure 1A). Longitudinal trends in cfDNA levels were explored in patients who had samples acquired at all study time points (n = 48). In these patients, peak cfDNA concentrations were detected at days 7 and 14 post-injury, with levels significantly higher than those recorded at days 1 and 3 post-burn (Figure 1B). CfDNA concentrations subsequently decreased, with the day 28 measurements significantly lower than those measured at days 7 and 14, and comparable to the concentrations recorded at days 1 and 3 (Figure 1B).

A positive correlation was identified between cfDNA concentrations and the number of operations patients had undergone since the timepoint of their previous sample donation (Supplementary Table S1). The measurements of cfDNA on day 28 positively correlated with age (r(n = 55) = 0.305, *p* = 0.024), although no other sampling timepoints demonstrated this relationship (Supplementary Table S2).

### 3.3. Comparison of Day One cfDNA Levels Measured in Serum Versus PFP

To date, the majority of studies that have measured cfDNA levels on the first day post-burn have analysed serum samples [20,21,33]. However, it has been reported that cfDNA concentrations measured in serum are higher than those detected in matched plasma samples [34,35]. To confirm this, we measured day 1 cfDNA concentrations in paired PFP and serum samples from burns patients (n = 20), who presented with a mean TBSA comparable to that analysed in previous studies [33]. We found that the median cfDNA concentrations in serum were significantly higher than those measured in plasma (Figure 1C).

### 3.4. Correlation of Day One cfDNA Levels with Clinical Indices

Day 1 cfDNA concentrations were analysed alongside clinical indices to explore associations between cfDNA and injury severity (Table 2). Positive associations were found between cfDNA concentrations and % TBSA (r(n = 95) = 0.413, *p* < 0.0005), full thickness % TBSA (r(n = 95) = 0.241, *p* = 0.021), Baux score (r(n = 95) = 0.347, *p* < 0.001), revised Baux score (r(n = 95) = 0.365, *p* = 0.0005), Denver score (r(n = 95) = 0.454, *p* < 0.0001), SOFA score (r(n = 95) = 0.391, *p* = 0.0005), and ABSI (r(n = 95) = 0.308, *p* = 0.003). Statistically significant negative correlations were identified between day 1 cfDNA concentrations and both hospital-free days (r(n = 95) = −0.387, *p* = 0.0002) and ITU-free days (r(n = 95) = −0.451, *p* < 0.0001, Table 2). A statistically significant positive correlation was found between day 1 cfDNA and ITU LOS r(n = 95) = 0.299, *p* = 0.004), although no such significance was found when correlating day 1 cfDNA and hospital LOS (r(n = 95) = 0.075, *p* = 0.469) (Supplementary Table S3).

### 3.5. Day 1 cfDNA Level Comparisons Between Clinical Outcome Subgroups

**Figure 2:**
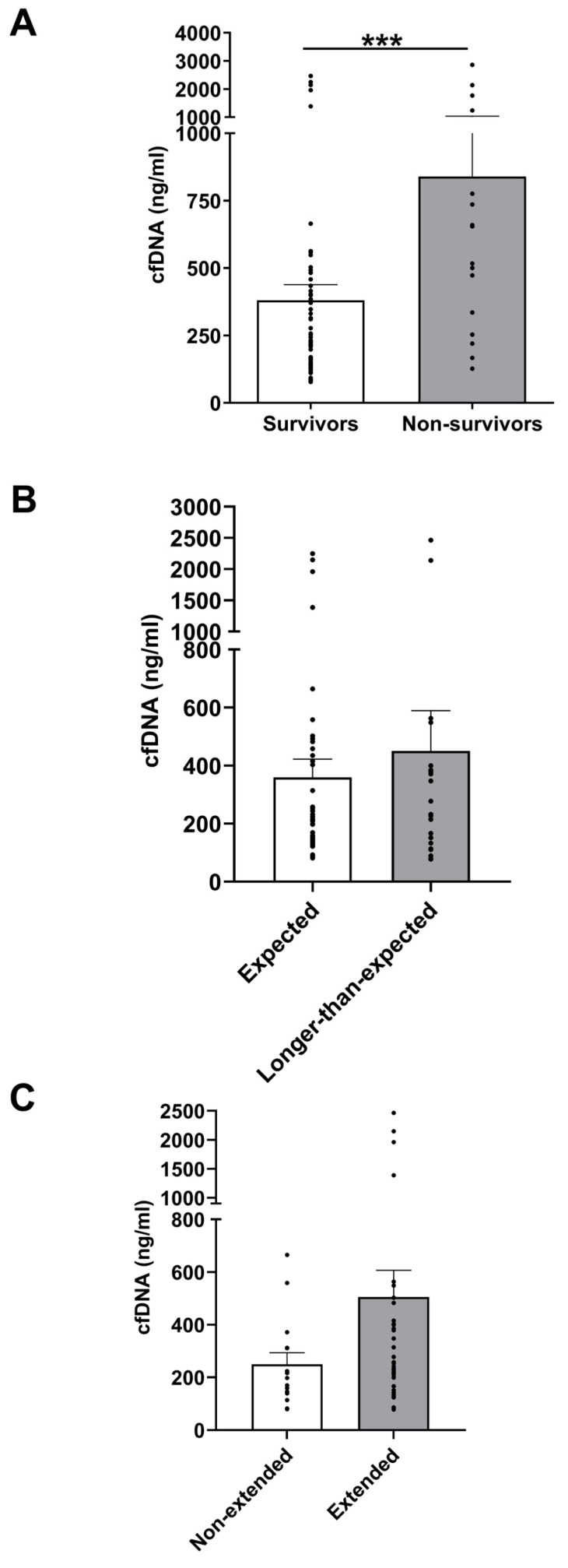
Circulating cell-free DNA (cfDNA) concentrations on day 1 of burn injury are significantly higher in non-survivors of major burn injuries. (**A**) CfDNA concentrations measured in survivors (n = 79) and non-survivors (n = 16) of major thermal injury. *** *p* < 0.0005. (**B**) CfDNA concentrations in burns patients with expected length of hospital stay (n = 56) and longer-than-expected hospital stay (n = 21). (**C**) CfDNA concentrations in burns patients with non-extended-ITU-length of stay (n = 15) and extended-ITU-length of stay (n = 38).

In an analysis that explored the association between cfDNA levels and mortality post-burn, day 1 cfDNA concentrations in PFP were compared between survivors (n = 79) and non-survivors (n = 16) (Supplementary Table S4). As shown in Figure 2A, on day 1 of injury, non-survivors presented with cfDNA concentrations that were significantly higher than those measured in the survivor group.

To facilitate a comparable analysis of LOS metrics, patients were stratified as per previous research [31] into expected-hospital-LOS (<2 days LOS/%TBSA) and longer-than-expected-hospital-LOS (≥2 days LOS/%TBSA) groups (Supplementary Table S5). There was no significant difference in cfDNA levels between patients in the expected-hospital-LOS and longer-than-expected-hospital-LOS groups (Figure 2B). Patients were also categorised into extended-ITU-LOS (≥10 days) and non-extended-ITU-LOS (<10 days) groups in accordance with previous work [32] (Supplementary Table S6), with no differences in cfDNA concentrations identified between these two groups (Figure 2C).

### 3.6. CfDNA as a Predictive Factor of Mortality Post-Burn

**Figure 3:**
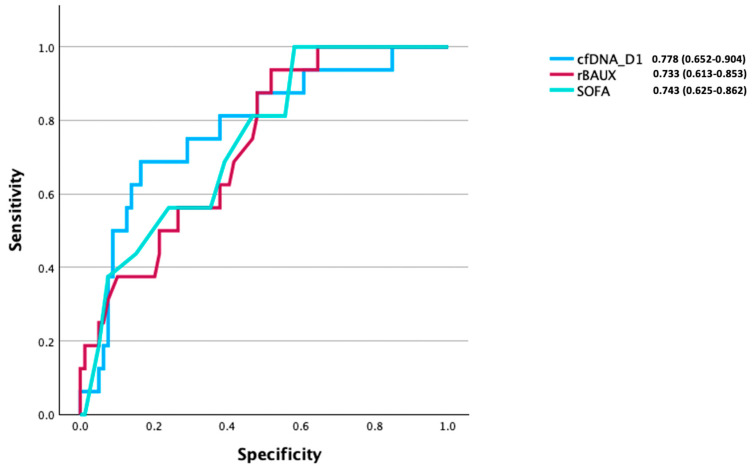
Area under the receiver operating curve (AUROC) analysis for mortality using the day 1 cell-free DNA (cfDNA) concentration, rBAUX score, and SOFA score. Figure label showing raw AUROC values (95% CI values) for all day 1 cfDNA values measured with recorded rBAUX and SOFA scores (n = 95).

After identifying differences in day 1 cfDNA concentrations between survivors and non-survivors (Figure 2A), an analysis was performed to explore the potential discriminatory ability of day 1 cfDNA to discern between these two groups (Figure 3). The area under the receiver operating curve (AUROC) analysis showed that day 1 cfDNA moderately discriminated between survivors and non-survivors (AUROC 0.778 (95% CI, 0.652–0.904)). An optimum cut-off value of 446.37 pg/mL cfDNA was calculated, which had a specificity of 0.70 and a sensitivity of 0.80. Day 1 cfDNA AUROC analysis by gender demonstrated no marked disparity in discriminatory ability between males (AUROC 0.802 (95% CI, 0.636–0.967)) and females (AUROC 0.756 (95% CI, 0.534–0.977)) (Supplementary Figure S2). AUROC models built on rBAUX and SOFA generated scores of 0.733 (95% CI, 0.613–0.853) and 0.743 (95% CI, 0.625–0.862), respectively.

### 3.7. IL-6 and IL-10 Levels in Burns Patients

**Figure 4:**
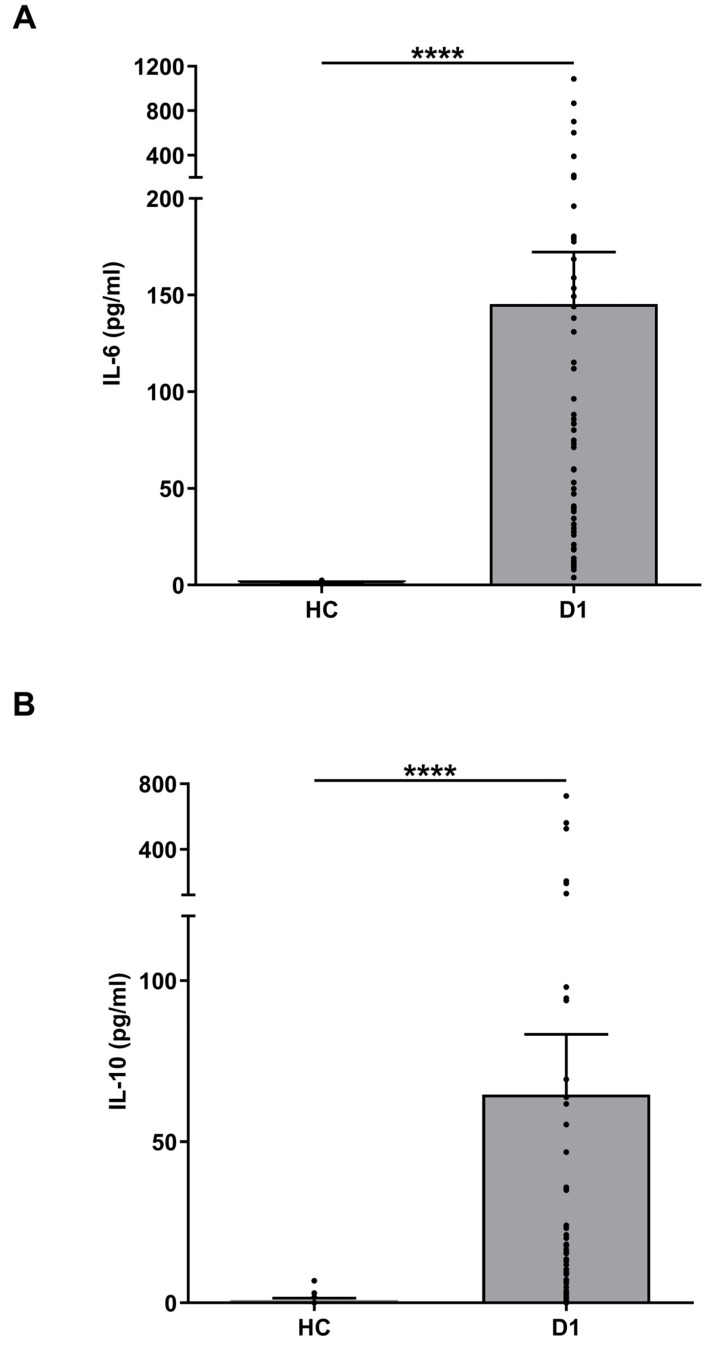
Major thermal injury results in elevated circulating concentrations of interleukin (IL)-6 and IL-10. (**A**,**B**) IL-6 (A) and IL-10 (**B**) concentrations measured in serum samples of healthy controls (HC, n = 13) and burns patients on day 1 post injury (IL = 6 n = 59, IL-10 n = 56). **** *p* < 0.0001.

**Figure 5:**
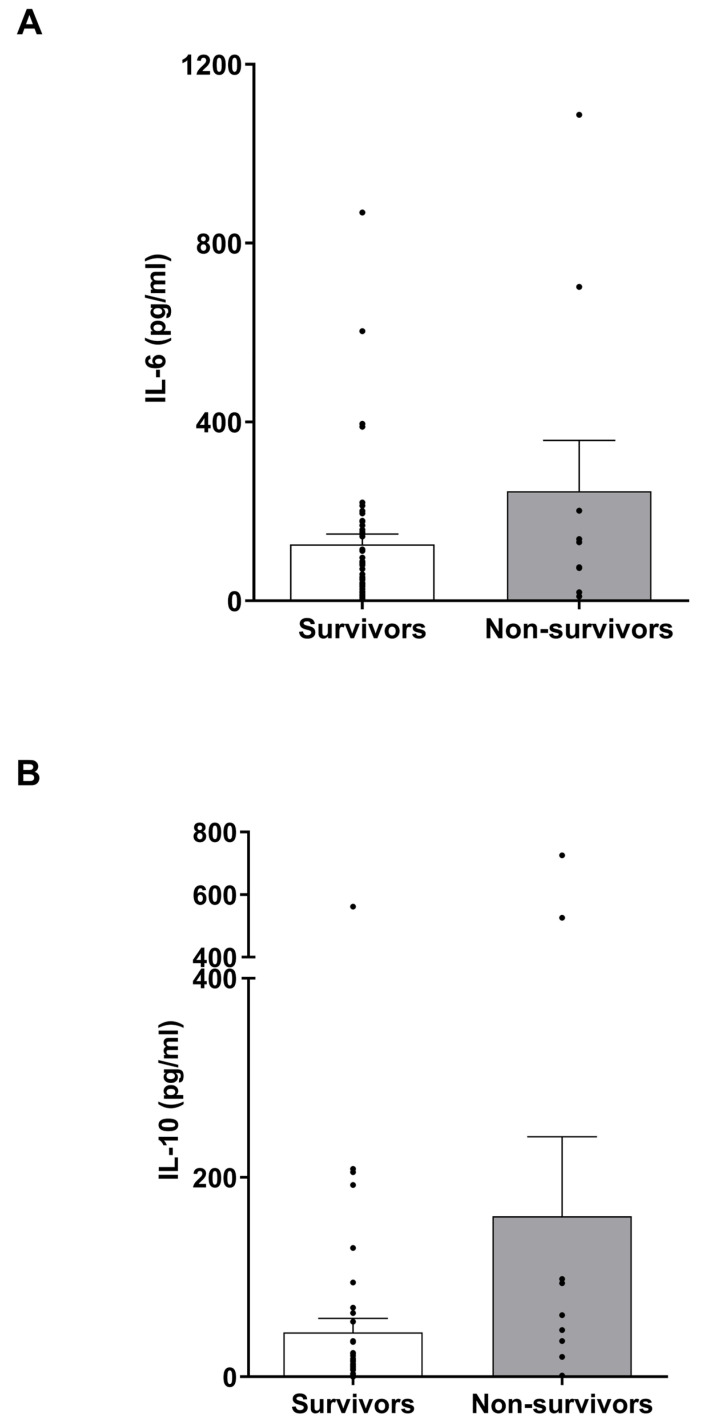
Circulating concentrations of interleukin (IL)-6 and IL-10 are comparable in survivors and non-survivors of major burns on day 1 of injury. (**A**) Comparison of serum IL-6 concentrations in survivors (n = 48) and non-survivors (n = 10) of major burns on day 1 of injury. (**B**) Comparison of serum IL-10 concentrations in survivors (n = 45) and non-survivors (n = 10) of major burns on day 1 of injury.

Previous studies have detected elevated concentrations of IL-6 and IL-10 in serum samples of non-survivors of burn injury when compared to survivors [27]. Although we observed a burn-induced increase in the circulating levels of both cytokines (Figure 4), a comparison of day 1 IL-6 and IL-10 levels between survivors and non-survivors (Supplementary Tables S7 and S8) revealed no significant differences in the concentration of either cytokine between these two groups (Figure 5).

### 3.8. Comparison of the Prognostic Utility of cfDNA, IL-6, and IL-10 Levels for Discriminating Between Survivors and Non-Survivors of Burn Injury

**Figure 6:**
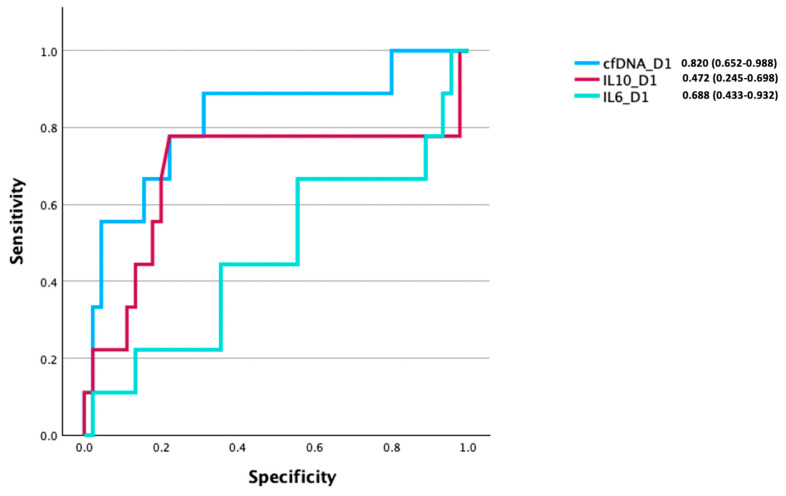
Area under the receiver operating curve (AUROC) analysis for mortality using day 1 cell-free DNA (cfDNA), interleukin (IL)-6 and IL-10 concentrations. Figure label showing raw AUROC values (95% CI values) for all patients with paired measures of day 1 cfDNA, IL-6 and IL10 concentrations (n = 54).

To investigate the relationship between cfDNA, IL-6, and IL-10 levels, and to further elucidate patient outcomes, we performed an AUROC analysis to compare their respective discriminatory abilities in discerning between survivors and non-survivors. This analysis was performed in all patients who had paired day 1 cfDNA, IL-6, and IL-10 measurements (n = 54). In this cohort, we found day 1 cfDNA levels to be a good predictor of mortality (AUROC 0.820 (95% CI, 0.652–0.988)). In comparison, models built on day 1 IL-6 (AUROC 0.472 (95% CI, 0.245–0.698)) or IL-10 (AUROC 0.688 (95% CI, 0.443–0.932)) concentrations exhibited poor discriminatory potential (Figure 6).

## 4. Discussion

Thermal injury remains a major form of trauma with the potential to cause profoundly negative patient clinical outcomes, including increased LOS, sepsis, and mortality [36,37]. These negative sequelae result from significant tissue destruction post-burn, with this driving subsequent inflammatory pathophysiology [38,39,40]. CfDNA is one factor released after tissue damage that has been explored in thermally injured patients, with associations identified between admission cfDNA levels and % TBSA [8,19,33], hospital LoS [19,20], and the severity of inhalational injuries [21]. Comparisons between cfDNA levels in patients experiencing partial-thickness and full-thickness burns have also been explored [20], with cfDNA proposed to have some efficacy in helping to predict survival post-burn [22]. However, this research was conducted on relatively small cohorts with limited insight into its time course or predictive potential [8,19,20,21,33]. Here, in a total cohort of 98 burns patients, we detected increased cfDNA plasma concentrations in patients relative to HC across the immediate (day 1) and acute (days 3–28) post-injury phases, with longitudinal measurements demonstrating a peak two weeks post-injury. Day 1 cfDNA levels positively correlated with clinical indices of injury severity, negatively correlated with LOS measures, and were markedly higher in non-survivors than in survivors. In turn, day 1 cfDNA levels showed good efficacy as a predictive marker of mortality. Taken together, these findings highlight the potential benefit of using cfDNA measurements as a prognostic aid in projecting the clinical progression of major burn patients.

Increased cfDNA has previously been reported in a series of studies conducted in smaller cohorts of burns patients [8,19,20,21,33]. However, half of these studies measured cfDNA concentrations in serum samples only [20,21,33]. Findings in non-burns patients have previously shown cfDNA to be increased in serum relative to plasma [34,35], possibly via neutrophil extracellular trap (NET) generation during the process of serum formation [41]. Consistently with this, our assessment of cfDNA levels in paired serum and plasma samples obtained on day 1 of injury revealed elevated concentrations in sera, indicating that differences in the type of sample chosen to measure cfDNA levels could explain the variation reported between studies [8,14,20]. Moreover, with the suggestion that immune cell activation during the clotting process could contribute to cfDNA release, we propose that plasma should be the biofluid of choice for cfDNA measurement, as the results generated are likely to reflect the tissue damage occurring post-injury and not be influenced by ex vivo production triggered during sample handling.

Only a minority of burns studies have explored time points beyond the first three days of admission, with one study measuring cfDNA on days 1, 3, 5, and 7 [22], another recording week 10 levels [8], and our own study, which measured cfDNA levels up to one-year post-injury [14]. This relative paucity of longitudinal cfDNA sampling has therefore left a notable post-burn time window that is uncharacterised. Our findings show a time course of elevated cfDNA at all timepoints measured post-injury in burn patients when compared to HC. This trend of longitudinal cfDNA level recording showed that the measured concentrations were significantly increased at day 7 and day 14, with peak levels at two weeks post-injury compared to those levels measured on days 1, 3, and 28. This progressive rise in cfDNA is suggestive of a secondary process, or processes, promoting additional DNA release, with the initial injury unlikely to cause such a temporal escalation of cfDNA concentrations in isolation. Such secondary insults could be the development of infection, systemic immune dysfunction (e.g., exaggerated NET production) and the early excision and grafting of burns, which are essential to achieving wound closure and optimising survival [42,43].

Indeed, surgical interventions have previously been shown to increase circulating cfDNA concentrations [44,45,46] with evidence of differential increases depending upon the scale of the surgical intervention that the patient was subjected to [44]. In line with this, in our cohort, we observed significant positive associations between plasma cfDNA levels and the number of surgical operations a patient underwent prior to sample acquisition. However, it is difficult to draw the conclusion that surgical interventions are the major driver of the elevations we observed in cfDNA concentrations in the sub-acute phase post-burn (days 7–14), given the significant thermal trauma experienced by our patients. This is in stark contrast to the abovementioned studies that quantified cfDNA levels before and after a single elective operation [44,45,46]. In this setting, an elective operative intervention represents a more controlled insult, with other influencing factors remaining relatively consistent. This makes the exact influence of surgery on circulating cfDNA levels difficult to account for in a trauma setting such as ours, where injury severity and the timing of operative intervention may vary. However, it would be folly not to recognise that the additional tissue damage caused by surgery, in addition to surgical dressing changes, will have contributed to the peak cfDNA levels we recorded at days 7 and 14 post-burn. The traumatic nature of our cohort’s injuries also means that there was a lack of pre-burn sampling, meaning that it was not possible for us to distinguish between burn- and co-morbidity-induced cfDNA release, or the role that medications may have played in cfDNA levels pre-injury. While we can rule out any influence glucocorticoid treatment may have had on plasma cfDNA levels (because patients on glucocorticoid therapy were not sampled as part of our study), excluding patients with comorbidities from our study would have impacted how representative our cohort of patients was in respect to resembling individuals who experience major burns. Indeed, by adhering to our specific study protocol, we excluded only those patients with established medical illnesses that would be expected to impact upon measures of systemic inflammation and/or immune function, such as individuals with HIV, hepatitis, and active malignancy.

A factor exacerbating post-burn triggers that result in DNA release is the significantly reduced activity of circulating DNAse that, we have reported, occurs for up to 28 days post-burn: an impairment that coincided with elevated cfDNA levels [14,47]. Our previous analyses of cfDNA in a smaller cohort of thermally injured patients interrogated its origins, with the results demonstrating the presence of elevated concentrations of nuclear but not mitochondrial-derived DNA in circulation [14]. Further investigations revealed the presence of citrullinated histone H3 (CitH3) within our cfDNA preparations [14]. As a specific marker of NET formation, the presence of CitH3 in samples collected during the acute post-injury phase (>7 days) demonstrates that NET release, induced during the systemic inflammatory response triggered by sterile surgical injury and/or pathogenic challenge, is a contributory factor in the persistent post-burn elevation of cfDNA [14].

Consistent with previous research [19], our results demonstrated an association between clinical indices of injury severity and day 1 cfDNA levels. Similarly, day 1 cfDNA concentrations positively correlated with % TBSA, a finding previously demonstrated in admission cfDNA levels measured in burns patients [8,19,33]. Significant positive correlations were also identified between day 1 cfDNA concentrations and other clinical indices—most notably, rBAUX and SOFA scores. Additionally, day 1 cfDNA negatively correlated with both hospital- and ITU-free days. This reemphasises previous findings that have described a positive relationship between the increased admission cfDNA concentrations and increased hospital LOS [19,20]. It also makes a significant contribution to the ongoing debate around the association between cfDNA and ITU LOS, with a range of conflicting earlier results previously detailed [19,20].

With our own data [14] and those of others [8] demonstrating that cfDNA concentrations are elevated at the time of hospital admission in burns patients, cfDNA could represent an early detectable circulating biomarker of poor clinical outcomes. With this in mind, we investigated the ability of cfDNA levels, measured on day 1 of burn injury, to predict patient mortality. The AUROC analysis indicated that day 1 cfDNA levels may be a good predictor of post-burn mortality, comparable to that found in trauma-based research [48], with an optimum cut-off value of 446.37 pg/mL exhibiting a sensitivity of 0.80 and specificity of 0.70. In both males and females, day 1 cfDNA levels were a good predictor of mortality, highlighting the potential utility of this approach in identifying patients, of both sexes, who are at risk of poor clinical outcomes. Mechanistically, the reported post-burn elevation in NET production [14,49] could contribute to mortality by perturbing blood flow in capillary networks, increasing the risk of multiple organ dysfunction and failure [50].

Due to the low mortality rate in our patient cohort, it was not possible for us to perform robust statistical testing to establish whether the AUROC value generated from the day 1 cfDNA measurements was significantly different to those AUROC scores calculated from models built on existing indicators of burn severity (rBAUX) and organ dysfunction (SOFA). Thus, from our data, we cannot decipher whether cfDNA measurements are a superior parameter by which to predict clinical outcomes post-burn. Future studies with larger patient cohorts and a higher mortality rate are therefore needed. Such investigations are important when considering the potential benefits that cfDNA measurements could offer in the clinical setting. For example, the calculation of SOFA scores is a time-consuming process that requires the assimilation of different haematological and clinical measures. Similarly, whilst rBAUX is a validated and widely accepted clinical scoring system that is reliant only on the summation of patient age, TBSA burns, and the presence/absence of inhalation injury [51], TBSA assessments are subjective and can be challenging even for an experienced surgeon [52], with incorrect estimations not infrequent [53] and high inter-rater variability regardless of the measurement tools used [54,55]. Thus, the rBAUX score may be affected by a degree of subjectivity. In contrast, cfDNA is obtainable from a single blood test on admission and is neither liable to estimation errors nor reliant on assimilating multiple laboratory/clinical measures, as per rBAUX and SOFA scores, respectively. Whilst we are not advocating for cfDNA measurements to be used in place of such validated scores in predicting the clinical outcomes of patients, we suggest that they could be considered as a useful adjunctive tool on first assessment in predicting mortality in burns patients. With this in mind, we are aware that the implementation of such a practice requires a number of factors to be considered, such as assay standardisation, cost, and turnaround time. To date, fluorescence-based assays have been the predominant method of choice for objectively measuring circulating cfDNA concentrations and assessing their relationship with patient outcomes in the context of burns [14,33], traumatic injury [18,48,56,57], and critical illness [58]. Importantly, these studies have commented upon the robustness, accuracy, reliability, speed, and cost effectiveness of this technique, with one study estimating the cost per test to be less than 1 USD [57]. Given that assay reliability and accuracy are of paramount importance for the prognostic utility of cfDNA measurements, we suggest that, moving forward, a standardised methodology for sample collection and processing is designed. This methodology would aim to minimise the effects that such pre-analytical variables as type of anti-coagulant, centrifugation speeds, storage temperature and number of freeze/thaw cycles would have on the quantification of cfDNA levels. Thinking longer term, with technological advancements, it is not unreasonable to propose that rapid point-of-care tests for the quantification of circulating cfDNA concentrations could be developed [59] and applied in the ITU of burns centres to aid in the rapid prognostication of patients and assist in decision making relating to treatment and management protocols.

Although our study is the largest to date exploring cfDNA in burns patients, further exploration of our findings in larger cohorts will no doubt be valuable in exploring its potential role in burn injury progression and prognosis. Our study also details the data collected in one burns centre alone; a multi-centric study with a more highly powered sample size may be able to better elucidate the use of cfDNA as a prognostic marker. This might allow for a more detailed characterisation of the usefulness of cfDNA when compared specifically to other established circulating biomarkers and clinical severity scores. Such future work would also benefit from being able to delineate the source of cfDNA. This would allow the longitudinal trends of cfDNA reported here to be characterised further, possibly identifying the specific causes of cfDNA elevations throughout the time course of burn patient care. A subtype analysis of cfDNA could then also be explored as markers of distinct clinical events, including the initial tissue insult, subsequent infection, and the impact of any surgical intervention.

## 5. Conclusions

Our findings show that major burns result in an immediate and persistent elevation in cfDNA. Furthermore, cfDNA concentrations were found to be higher on day 1 post-burn in non-survivors, with day 1 cfDNA showing efficacy as a prognostic marker of mortality. This research suggests that cfDNA represents a rapid objective measure that could have utility in predicting mortality as part of acute burn patient assessment.

## Figures and Tables

**Table 1:** Burn patients’ demographics.

Characteristic	Burns Patients (n = 98)
Age, years	47 (16–84)
Gender (M:F)	77:21
% TBSA	35 (15–85)
% FT TBSA	19 (0–80)
Inhalation injury (Y:N)	44:54
Mechanism of injury:	
*Flash*, n *(%)*	7 (7)
*Flame*, n *(%)*	82 (84)
*Flame and flash*, n *(%)*	5 (5)
*Electrical*, n *(%)*	1 (1)
*Scald*, n *(%)*	3 (3)
Co-morbidities:	
*Respiratory*, n *(%)*	12 (12)
*Cardiovascular*, n *(%)*	15 (15)
*Endocrine/metabolic*, n *(%)*	12 (12)
*Psychiatric*, n *(%)*	30 (31)
*Neurological*, n *(%)*	10 (10)
*Gastrointestinal*, n *(%)*	3 (3)
*Musculoskeletal*, n *(%)*	4 (4)
ABSI	7 (2–14)
Baux	82 (34–143)
rBaux	90 (39–160)
Day 1 SOFA	7 (0–17)
Day 1 Denver	2 (0–7)
ITU-free days	15 (0–30)
Hospital-free days	4 (0–20)
Mortality (Y:N)	17:81

Data are expressed as the mean (range) unless otherwise stated.

**Table 2:** Correlative analysis investigating the relationships between circulating cell-free DNA (cfDNA) concentrations on day 1 of thermal injury and clinical indices.

	Correlation (R)	Significance (*p*)	FDR Adjusted *p*-value
TBSA % (n = 95)	0.413	**<0.0001**	**0.0001**
TBSA FT (n = 95)	0.241	**0.019**	**0.021**
Baux (n = 95)	0.347	**<0.001**	**0.0009**
rBaux (n = 95)	0.365	**<0.0005**	**0.0005**
Denver (n = 95)	0.454	**<0.0001**	**<0.0001**
SOFA (n = 95)	0.391	**<0.0001**	**0.0002**
ABSI (n = 95)	0.308	**0.002**	**0.003**
Hospital-free days (n = 95)	−0.387	**0.0003**	**0.0002**
ITU-free days (n = 95)	−0.451	**<0.0001**	**<0.0001**
Hospital LOS (n = 95)	0.075	0.469	0.469
ITU LOS (n = 95)	0.299	**0.003**	**0.004**

## Data Availability

The raw data supporting the conclusions of this article will be made available by the authors, without undue reservation.

## Supplementary Materials

The following supporting information can be downloaded at: https://www.mdpi.com/article/10.3390/cells14110821/s1, Table S1. Correlative analyses examining the relationship between plasma cell-free DNA (cfDNA) concentrations at days 3–28 post burn injury and the total number of operations (NoO) that occurred in the time period since the previous cfDNA measurement. Table S2. Correlative analyses examining the relationship between patient age and plasma cell-free DNA concentrations in thermally injured patients across days 1–28 post-injury. Table S3. Correlative analyses examining the relationship between intensive care unit length of stay, hospital length of stay, and plasma cell-free DNA concentrations in thermally injured patients across days 1–28 post-injury. Table S4. Demographic and clinical data of survivors and non-survivors of thermal injury from whom measurements of plasma cell-free DNA levels were obtained on day 1 of burn injury. Table S5. Demographic and clinical data of thermally injured patients with expected and longer-than-expected lengths of hospital stay from whom measurements of plasma cell free DNA levels were obtained. Table S6. Demographic and clinical data of thermally injured patients with extended and non-extended intensive treatment unit (ITU) lengths of stay from whom measurements of plasma cell-free DNA levels were obtained. Table S7. Demographic and clinical data of survivors and non-survivors of thermal injury from whom measurements of plasma interleukin-6 levels were obtained. Table S8. Demographic and clinical data of survivors and non-survivors of thermal injury from whom measurements of plasma interleukin-10 levels were obtained. Figure S1. Flow diagram showing patient recruitment and cfDNA analysis in the study. Figure S2. Area under the receiver operating curve (AUROC) analysis for mortality using day 1 cell-free DNA (cfDNA) for male (A, n = 74) and female (B, n = 21) patients. Figure S3. Area under the receiver operating curve (AUROC) analyses for mortality using day 1 cfDNA (A), SOFA (B), rBAUX (C), IL-10 (D) and IL-6 (E).
