# Supplementary material for: Day One Cell-Free DNA Levels as an Objective Prognostic Marker of Mortality in Major Burns Patients
Source: Cells. 2025 Jun 1;14(11):821. doi: 10.3390/cells14110821 (PMC12155140; doi:10.3390/cells14110821)
Supplement: Supplementary file 1 [file cells-14-00821-s001.zip › supplementary figures.pdf]

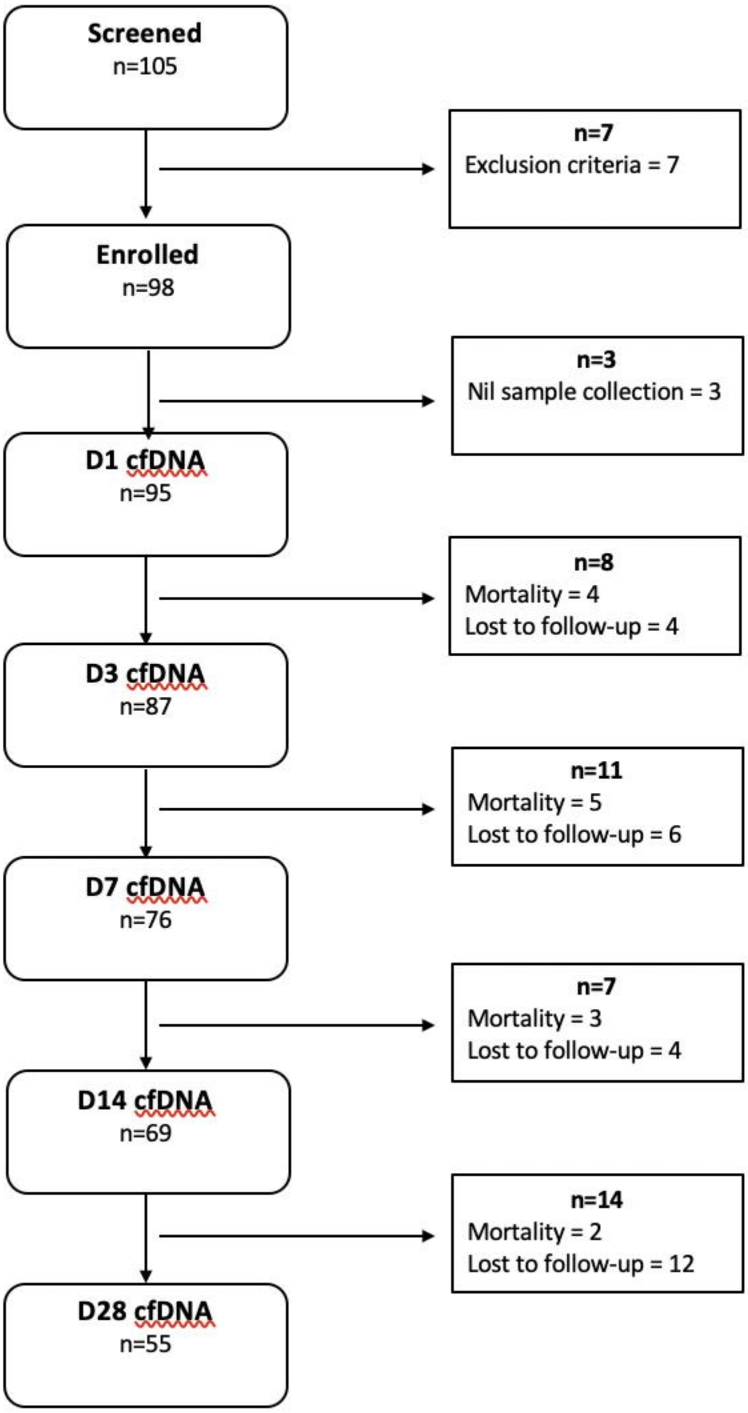

Supplementary Figure S1. Flow diagram showing patient recruitment and cfDNA analysis in study. Patients excluded were in line with study protocols. Mortality refers to those who died prior to collection of the next sampling time. *Lost to follow-up* refers to those patients for whom samples were unavailable for analysis due to refusal of sampling, study withdrawal or acquisition of insufficient blood volume to allow for the preparation of plasma for cfDNA analysis.

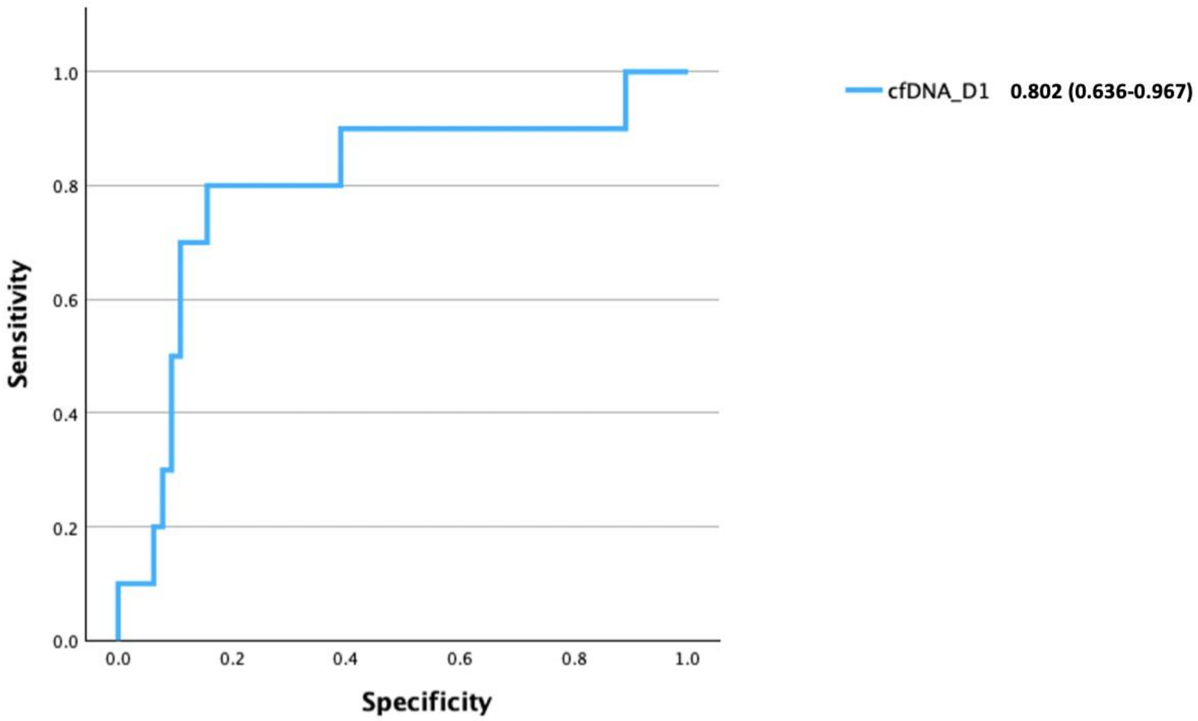

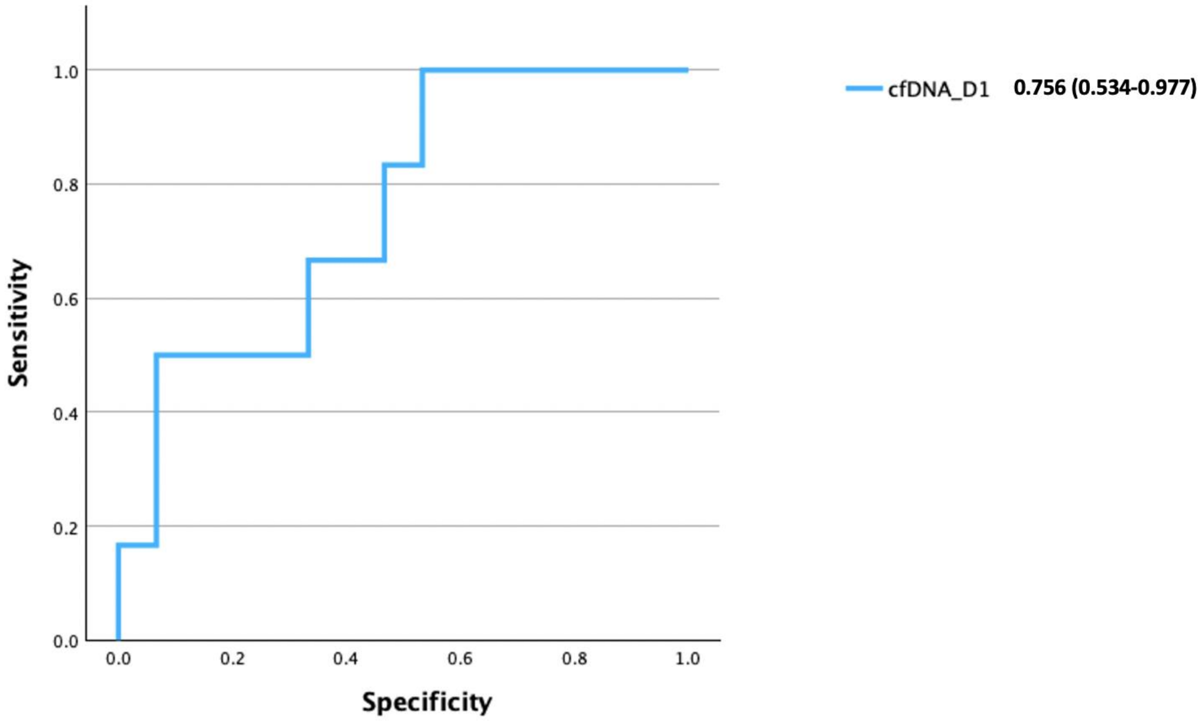

Supplementary Figure S2. Area under the receiver-operating curve (AUROC) analysis for mortality using day 1 cell free DNA (cfDNA) for male (A, n=74) and female (B, n=21) patients. Figure labels show raw AUROC values (95% CI values).

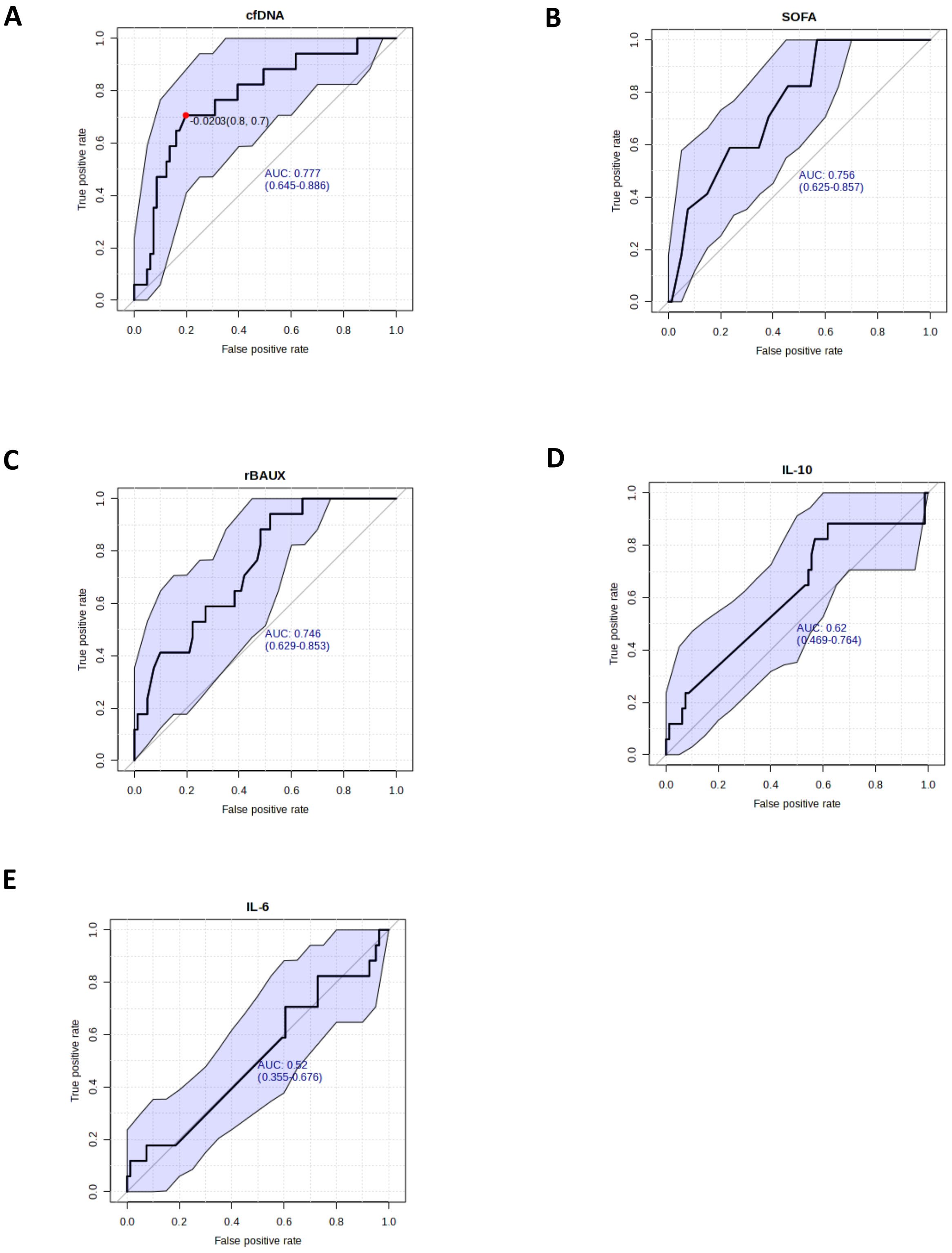

Supplementary Figure S3. Area under the receiver-operating curve (AUROC) analyses for mortality using day 1 cfDNA (A), SOFA (B), rBAUX (C), IL-10 (D) and IL-6 (E). Figures show raw AUROC values (95% CI values). Confidence intervals are plotted for each ROC. Optimal cut-off value plotted on cfDNA ROC in (A) for sensitivity of 0.80 and specificity of 0.70, corresponding to 446.37 pg/ml cut-off.
