# Supplementary material for: Day One Cell-Free DNA Levels as an Objective Prognostic Marker of Mortality in Major Burns Patients
Source: Cells. 2025 Jun 1;14(11):821. doi: 10.3390/cells14110821 (PMC12155140; doi:10.3390/cells14110821)
Supplement: Supplementary file 1 [file cells-14-00821-s001.zip › supplementary tables.pdf]

Supplementary Table S1. Correlative analyses examining the relationship between plasma cell free DNA (cfDNA) concentrations at days 3-28 post burn injury and the total number of operations (NoO) that occurred in the time period since the previous cfDNA measurement.

	**Day 3**	**Day 7**	**Day 14**	**Day 28**
NoO D1-3	**R=0.214<br>(-0.002-0.412)<br>p=0.046<br>n=87**	-	-	-
NoO D3-7	-	**R=0.532<br>(0.342-0.680)<br>p=<0.001<br>n=76**	-	-
NoO D7-14	-	-	**R=0.712<br>(0.566-0.814)<br>p=<0.001<br>n=69**	-
NoO D14-28	-	-	-	**R=0.668<br>(0.483-0.796)<br>p=<0.001<br>n=55**

95% confidence intervals are presented in parentheses. Analysis was performed by Spearman's rank correlation coefficient. Significant associations are indicated in bold font.

Supplementary Table S2. Correlative analyses examining the relationship between patient age and plasma cell free DNA concentrations in thermally-injured patients across days 1-28 post-injury.

	**Day 1**	**Day 3**	**Day 7**	**Day 14**	**Day 28**
Age	R= 0.100<br>(-0.114-0.297)<br>p= 0.355<br>n= 95	R=0.196<br>(-0.022-0.396)<br>p=0.069<br>n=87	R=0.128<br>(-0.107-0.350)<br>p=0.269<br>n=76	R=0.121<br>(-0.126-0.354)<br>p=0.323<br>n=69	**R=0.305**<br>**(0.035-0.533)**<br>**p=0.024**<br>**n=55**

95% confidence intervals are presented in parentheses. Analysis was performed by Spearman's rank correlation coefficient. Significant associations are indicated in bold font.

Supplementary Table S3. Correlative analyses examining the relationship between Intensive care unit length of stay, hospital length of stay and plasma cell free DNA concentrations in thermally-injured patients across days 1-28 post-injury.

	**Day 1**	**Day 3**	**Day 7**	**Day 14**	**Day 28**
ITU LOS	**R= 0.299 (0.100-0.477) p= 0.0040 n=95**	**R=0.499 (0.317-0.646) p=<0.0001 n=87**	**R=0.682 (0.535-0.789) p=<0.0001 n=76**	**R=0.805 (0.698-0.877) p=<0.0001 n=69**	**R=0.700 (0.528-0.817) p=<0.0001 n=55**
Hospital LOS	R= 0.075 (-0.134-0.278) p= 0.469 n= 95	**R=0.273 (0.060-0.463) p=0.010 n=87**	**R=0.381 (0.164-0.564) p=0.0007 n=76**	**R=0.588 (0.402-0.727) p=<0.0001 n=69**	**R=0.476 (0.234-0.663) p=0.0002 n=55**

95% confidence intervals are presented in parentheses. Analysis was performed by Spearman's rank correlation coefficient. Significant associations are indicated in bold font. ITU, Intensive Care Unit; LOS, Length of Stay

Supplementary Table S4. Demographic and clinical data of survivors and non-survivors of thermal injury from whom measurements of plasma cell free DNA levels were obtained on day 1 of burn injury.

**Characteristic**	**Survivors (n=79)**	**Non-survivors (n=16)**	**p**
Age, years	44 (16-83)	57 (22-83)	**0.003**
Gender (M:F)	64:15	10:6	0.104
% TBSA	34 (15-80)	45 (15-85)	**0.046**
% FT TBSA	16 (0-75)	32 (0-80)	**0.004**
Inhalation injury (Y:N)	35:44	8:8	0.676
ABSI	7 (2-13)	10 (5-14)	**0.001**
Baux	77 (34-130)	102 (72-143)	**0.001**
rBaux	85 (39-147)	111 (72-160)	**0.003**
Day 1 SOFA	6 (0-17)	10 (4-15)	**0.001**
Day 1 Denver	2 (0-7)	4 (1-7)	**0.001**
ITU-free days	18 (0-30)	5 (0-30)	**<0.0001**
Hospital-free days	5 (0-24)	0 (0)	**0.003**

Data are expressed as mean (range) unless otherwise stated.

Supplementary Table S5. Demographic and clinical data of thermally-injured patients with expected and longer than expected lengths of hospital stay from whom measurements of plasma cell free DNA levels were obtained.

**Characteristic**	**Expected hospital length of stay (n=56)**	**Longer than expected hospital length of stay (n=21)**	**p**
Age, years	42 (17-83)	48 (16-79)	0.144
Gender (M:F)	52:4	10:11	**<0.0001**
% TBSA	32 (15-80)	35 (15-75)	0.355
% FT TBSA	12 (0-71)	24 (0-75)	**0.002**
Inhalation injury (Y:N)	20:36	13:8	**0.039**
ABSI	7 (2-13)	7 (3-13)	0.310
Baux	74 (39-130)	83 (34-125)	0.100
rBaux	80 (39-147)	94 (50-142)	0.052
Day 1 SOFA	5 (0-15)	10 (1-17)	**0.0002**
Day 1 Denver	1 (0-7)	3 (0-6)	**0.001**
ITU-free days	21 (0-30)	10 (0-30)	**0.0002**
Hospital-free days	6 (0-20)	0 (0-0)	**<0.0001**

Data are expressed as mean (range) unless otherwise stated.

Supplementary Table S6. Demographic and clinical data of thermally-injured patients with extended and non-extended intensive treatment unit (ITU) lengths of stay from whom measurements of plasma cell free DNA levels were obtained.

**Characteristic**	**Non-extended ITU length of stay (n=15)**	**Extended ITU length of stay (n=38)**	**p**
Age, years	42 (20-79)	42 (16-72)	0.965
Gender (M:F)	12:3	31:7	0.895
% TBSA	24 (15-47)	44 (18-80)	**<0.0001**
% FT TBSA	6 (0-28)	28 (0-75)	**<0.0001**
Inhalation injury (Y:N)	5:10	28:10	**0.006**
ABSI	6 (3-9)	8 (2-13)	**0.020**
Baux	67 (39-96)	86 (34-130)	**0.016**
rBaux	72 (39-100)	98 (40-147)	**0.002**
Day 1 SOFA	4 (0-10)	10 (2-17)	**0.0001**
Day 1 Denver	1 (0-4)	3 (1-7)	**<0.0001**
ITU-free days	25 (17-29)	7 (0-20)	**<0.0001**
Hospital-free days	7 (0-20)	1 (0-11)	**<0.0001**

Data are expressed as mean (range) unless otherwise stated.

Supplementary Table S7. Demographic and clinical data of survivors and non-survivors of thermal injury from whom measurements of plasma interleukin-6 levels were obtained.

Characteristic	Survivors (n=48)	Non-survivors (n=10)	p
Age, years	45 (19-79)	59 (22-83)	**0.016**
Gender (M:F)	38:10	7:3	0.527
% TBSA	34 (15-80)	40 (15-80)	0.390
% FT TBSA	18 (0-75)	27 (0-80)	0.138
Inhalation injury (Y:N)	18:30	4:6	0.882
ABSI	7 (2-13)	10 (6-14)	**0.006**
Baux	79 (42-130)	99 (72-143)	**0.018**
rBaux	85 (45-147)	106 (72-160)	0.051
Day 1 SOFA	6 (0-17)	10 (4-14)	**0.005**
Day 1 Denver	2 (0-6)	3 (1-6)	**0.004**
ITU-free days	20 (0-30)	3 (0-30)	**<0.0001**
Hospital-free days	6 (0-24)	0 (0)	**0.019**

Data are expressed as mean (range) unless otherwise stated.

Supplementary Table S8. Demographic and clinical data of survivors and non-survivors of thermal injury from whom measurements of plasma interleukin-10 levels were obtained.

**Characteristic**	**Survivors (n=45)**	**Non-Survivors (n=10)**	**p**
Age, years	45 (19-79)	59 (22-83)	**0.01**
Gender (M:F)	36:9	7:3	0.489
% TBSA	35 (15-80)	40 (15-80)	0.521
% FT TBSA	19 (0-75)	27 (0-80)	0.189
Inhalation injury (Y:N)	18:27	4:6	1.000
ABSI	7 (2-13)	10 (6-14)	**0.008**
Baux	79 (42-130)	99 (72-143)	**0.023**
rBaux	86 (45-147)	106 (72-160)	0.066
Day 1 SOFA	6 (0-17)	10 (4-14)	**0.006**
Day 1 Denver	2 (0-6)	3 (1-6)	**0.009**
ITU-free days	20 (0-30)	3 (0-30)	**0.0001**
Hospital-free days	6 (0-24)	0 (0)	**0.024**

Data are expressed as mean (range) unless otherwise stated.
